# High-quality development of virtual reality industry from the perspective of environmental regulation

**DOI:** 10.3389/fpsyg.2022.1005585

**Published:** 2022-10-12

**Authors:** Liping Zhang, Yi Yang, Kaiqi Xiong

**Affiliations:** School of Economics and Management, East China Jiaotong University, Nanchang, China

**Keywords:** high-quality development, virtual reality industry, environmental regulation, green development, structural equation model

## Abstract

From the perspective of environmental regulation, this paper constructed a high-quality development evaluation system for the virtual reality industry (hereinafter referred to as VRI), calculated the high-quality development level of the VRI, and empirically studied the path of high-quality development of VRI based on the research methods of entropy weight method and structural equation model (hereinafter referred to as SEM), as well as the data of listed companies of VRI in China from 2011 to 2020. The results showed that (1) VRI adapted to the high-quality development level, but there was a certain gap, its high-quality development degree shows a rising trend; (2) the degree of corporate public welfare development had a positive effect on corporate profitability, while the degree of green development and coordinated development had a weak impact on corporate profitability; (3) open and innovative dimensions had a reverse effect on the profitability of enterprises. Enterprises should seek ways to reduce environmental costs and enhance competitiveness. The industrial chain, technology chain and value chain should be constructed to promote the healthy development of VRI and provide support for the formulation of high-quality development policies for VRI.

## Introduction

Global economies have been growing quickly since the 1960s in order to achieve the transition from increasing quantity to improving quality. Quality has become the key point and core element of national social construction. By the 1990s, innovation had become crucial to the global new economy and development boom. As a high-end product of industrial digitization, the main difference between VRI and traditional industries lies in the application and innovation ability of digital technology (Zhang, 2016). Most of the traditional industries are capital intensive and labor-intensive industries (Wang et al., 2016), which are highly replaceable and vulnerable to external fluctuations. However, most of the VRI are knowledge intensive and technology intensive industries, which are industries with large growth potential and high comprehensive benefits. The products and services they produce have high added value and high technology (Can and Gozgor, 2018), and play a major leading role in the long-term development of the economy and society (Wu and Zhang, 2017). Companies that can integrate VR technology into the core aspects of production and operation will have a greater voice in future, according to Michael, the head of Facebook’s VR team in the USA, who stated in 2017 that the development of VR would bring disruptive impact and reshape the industry chain pattern. VR technology is centered on technological capabilities, and then generates a multi-dimensional network system and organic ecosystem with the driving force, competitiveness, and industrial sustainability characteristics (Shen et al., 2020). Currently, the VRI is maximally applied in various fields such as medicine, media, and teaching (Pontonnier et al., 2014; Guo and Yi, 2020; Guo et al., 2020). The cognitive exploration of “VR+” upgrading has been carried out, showing that VR technology has a linkage effect and can help transform and upgrade other industries. It is clear that the VRI is crucial to advancing the digital economy and high-quality economic development; however, the COVID-19 is still having a significant negative impact on most of the world’s economies (Yang and Wang, 2021; Shi and Li, 2022), and the economic prospects is extremely uncertain. Additionally, some VR companies are still stuck in content production due to a lack of core competitiveness and a low-end homogenization phenomenon (Dai, 2017; Gao, 2017). If the VRI is unable to produce new products, it will not be able to sustain itself, few of them have been combined with VRI specific studies. This is an urgent issue for all types of enterprises as the economy enters a phase of high-quality development.

The VRI is not a closed and isolated system, and there is a close interconnection between the industry and the environment (Guo et al., 2020). Environmental regulation can promote high-quality development of China’s economy by adjusting industrial structure and innovation input (Ge, 2020). Therefore, environmental regulation is a good entry point when trying to solve the issue. At present, there is no unanimous conclusion on the relationship between environmental regulation and high-quality industrial development, and the main contradiction lies in the higher compliance cost and innovation compensation (Li and Li, 2017; Huang et al., 2018; Li et al., 2019), but according to the “strong Porter hypothesis” in most cases, the innovation effect brought by reasonable environmental regulation will fully compensate for the cost effects. In addition, from the perspective of both quantity and quality of economic growth, environmental regulation has a significant inhibitory effect on the quantity of economic growth, while it has a significant promotional effect on the quality of economic growth (Huang and Gao, 2016; Wang and Lu, 2018). Green trade openness and level of the knowledge-skills of the society shows a significant and positive effect on human well-being across quantiles. Environmental regulation through the development of environmental regulatory tools such as green entry barriers will stimulate enterprises to improve their innovation capacity, improve the efficiency and quality of green product production, and promote the transformation and upgrading of industrial structure (Li and Nie, 2009; Yu and Wu, 2022). From the environmental regulation perspective, there are still research gaps on whether the VRI can maintain long-term competitive advantages and achieve high-quality development. Therefore, this paper focuses on exploring the following issues based on the environmental regulation perspective to fill the gap in the research on the influencing factors of VRI’s high-quality development and the level of VRI’s high-quality development.

Q1: Does VRI adapt to high-quality development?

Q2: What stage is the VRI’s high-quality development at right now?

Q3: What is the mechanism of high-quality development of VRI in China?

The VRI, as a conceptual high-tech industry, has a natural coupling with innovative development and green development, and the industrial environment is more conducive to the benefits of collaborative development, open development, and shared development. This study builds an evaluation system for high-quality development levels predicated on the theory of environmental regulation, chooses panel data of listed companies in China for VR concepts from 2011 to 2020, and uses the entropy method to measure the high-quality development level of the VRI. The SEM is combined to examine the path and evolutionary mechanism of the high-quality development of China’s VRI, with a view to enriching the theoretical framework of high-quality development, more effectively direct pertinent enterprises to carry out pertinent innovative practices, and serve as a helpful guide for China’s economic development practices.

The following are the study’s contributions: first, incorporating environmental regulation theory into the variable model, analyzing the level of high-quality development from the perspective of environmental regulation, and using the high-quality development level measure to investigate the applicability of the VRI to various industries. Second, the entropy method combined with the SEM is used to investigate the relationship between the VRI and high-quality development. Third, it deconstructs the channels of high-quality development influence and analyzes the mechanism of the effect of high-quality development on the operational capability of the VRI. The study also demonstrates that public welfare development has a greater influence on the VRI, yielding more focused conclusions that provide theoretical support and empirical directions for future research on high-quality VRI development.

The subsequent parts of this paper are organized as follows: Part II theoretical analysis and derive research hypotheses from them for empirical testing; Part III research design, data processing, and VRI’s high-quality development level measurement; Part IV empirical analysis, model fit test and further discussion of the impact path of high-quality development on VRI’s profitability; Part V, summarize the paper’s research findings and discussion; Part VI, put forward policy recommendations.

## Theoretical analysis and hypothesis

This paper attempts to analyze the level of high-quality development of VRI and the path of high-quality development on the profitability of VRI from the perspective of environmental regulation, which can be distinguished into two interrelated issues: first, the coupling degree of VRI and high-quality development; second, the impact and transmission mechanism of high-quality development on the profitability of VR enterprises.

### The coupling degree of virtual reality industry and high-quality development

Environmental regulation is highly linked to both high-quality development and sustainable development goals (Hereinafter referred to as SDG). Innovative development is an important means for enterprises to reduce environmental costs, and the results of innovation can, in turn, increase the willingness of cooperation among enterprises and further accelerate the achievement of low pollution and low emissions. In the digital economy era, the development model driven by innovation and supported by openness is the mainstream, and digitization is the core element to promote high-quality economic development (Zhao et al., 2020). The cultivation and development of the VRI, as a new variable of economic quality and efficiency, will strongly promote the reform and innovation of science and technology systems, enhance innovation capacity, and promote the innovation-driven strategy. As the main driving force in coordinating the contradiction between economic development, resources, and the environment, and promoting regional greening, innovation has an emission reduction effect and can suppress urban environmental pollution (Fang and Yang, 2021), and plays an important role in promoting high-quality development oriented by green concepts, so the innovation-driven development strategy has long-term and significant significance for the green economic development (Zou and Liao, 2017). In contrast, environmental regulation policies do not affect the green transformation of industries through technological upgrading, and it is difficult to achieve immediate effects of green development strategies on corporate innovation in the short term (Yang and Zhou, 2022). As a result, this study temporarily excludes the impact of green development on innovation development and proposes the following hypothesis:

*H1*: The relationship between green development and innovative development is influenced positively.

Open development means enabling enterprises to access social networks and expand channel relationships. With the rapid development of mobile internet and digital technology, the digital economy has emerged as the world’s future development direction, serving as an important kinetic energy to promote global economic growth. Artificial intelligence, big data, cloud computing, blockchain, 5G, and other new information technology can be used to realize changes in production, manufacturing, sales, and other economic factors, build a green low-carbon technology system with science and technology innovation as the driving force, and stimulate industrial innovation (Liu et al., 2021). Through technological innovation in open conditions, the VRI can accelerate the sense of urgency, mission, and responsibility of independent innovation, play the characteristics of strong VR rendering effect, promote a high level of openness, and industry integration as the main line, enrich VR business model, integrate VRI chain resources, and build a development pattern of the domestic and international double cycle (Chi, 2021). At the same time, some scholars have also found that Chinese OFDI only has a reverse spillover effect on the ability to imitate innovation domestically, while it has a suppressive effect on the ability to innovate both independently and secondarily. This ultimately leads to open development instead having a dampening effect on overall innovation capacity, but the effects at all levels are statistically insignificant (Xie et al., 2014). As a result, this study focuses only on the role of innovation development in influencing open development and proposes the following hypothesis:

*H2*: The relationship between innovative development and open development is influenced positively.

Collaborative development embodies the process of building trust. A new round of technological revolutions and industrial change is on the horizon, and digital technology has a massive multiplier effect. As a realistic direct productivity, it plays an important role in integrating factors such as policies, industries, talents, capital, and so on. Through osmosis, the innovation and entrepreneurship ecosystem formed by each innovation factor can enable collaborative development, thus improving society’s overall productivity level. Technology innovation, as a new growth point for economic development, includes fostering strategic emerging industries and high-tech industrialization, as well as through the “two-wheel drive” of science and technology innovation and institutional innovation (Zhang YY, 2020), the industry-university-research collaborative innovation supported by scientific innovation and market-oriented will be realized, and the integration of industrialization, informatization and synergy will be promoted. On the other hand, the role of collaborative development on innovation development is reflected in the fact that the development of innovation activities requires close personnel exchange and collaboration with processing and manufacturing activities, and the relocation of processing and manufacturing links will cause damage to the industrial commons, thus affecting the effective enhancement of innovation capacity (Pisano and Shih, 2009). As a result, the following hypothesis is proposed in this study:

*H3*: The relationship between innovative development and collaborative development is influenced positively.

Green development, from the standpoint of environmental regulation, necessitates enterprises having a complete understanding of potential environmental resource consumption, which is the most basic goal of environmental regulation. In response to global warming and ecological degradation, the European Union proposed carbon reduction reforms, the USA proposed carbon reduction policies, and China set a “double carbon” target, with carbon peaking and carbon neutral as the grasping hand, to begin a comprehensive and deep green low-carbon systemic change (Ren and Song, 2020), indicating that green should be the distinctive color of high-quality development. Green low-carbon development is a resource-saving and environmentally friendly development model that, on the one hand, focuses on energy conservation and emission reduction to improve ecological civilization construction and, on the other, uses green low-carbon technologies to promote economic globalization in the direction of universal supply and develop a higher-level open economy (Guo, 2021). In contrast, opening up to the outside world will instead have a dampening effect on green development. Although the initial opening up to the outside world will promote the growth of green GDP, with higher levels of opening up to the outside world will have a dampening effect on green economic development (Lu et al., 2018), the opening up to the outside world is also inhibitory to a certain extent to the development of the green economy in different regions of China (Sun et al., 2014; Xue and Wang, 2021). As a result, this study does not consider the impact of open development on green development, and proposes the following hypothesis:

*H4*: The relationship between green development and open development is influenced positively.

Recently, a new wave of technological and industrial changes, represented by information technology and cloud computing, has emerged to help improve and upgrade the new digital tools and market them, as well as upgrade the support empowerment capacity on the supply side of digital transformation (Cai, 2021), which in turn promotes the country’s, industry’s, and enterprises’ own common development. Digital technology and the VRI evolve in tandem as part of a national strategy of data integration and open sharing, adjusting the industrial structure from the supply and demand sides, increasing the realistic need to inject new impetus into global economic growth, fully utilizing the open context of global digital economy cooperation, focusing on the sharing of development results, and prompting countries to cooperate and share the dividends of the times (Xu and Yang, 2021). Regarding the role of shared development on open development, some scholars have also proposed that uncontrolled external knowledge sharing will lead to accidental knowledge leakage and reduce the radical innovation performance of enterprises, which is not conducive to attracting investment from multinational companies (Ritala et al., 2018). This study uses public welfare as an alternative to shared development, and proposes the following hypotheses:

*H5*: The relationship between open development and public welfare is influenced positively.

Shared development is an important means of increasing the number of environmental resources. On the one hand, the “Internet+” trend has changed market demand and consumption habits from physical consumption to online consumption, in which “cooperative consumption,” as a new way of sharing economy, integrates and optimizes innovative resources such as through network tools and Internet technology. The emergence of shared bicycles and shared rechargeable batteries has formed a new business operation model to optimize institutional innovation, build a healthy financing platform, improve regulatory effectiveness, ensure trust mechanisms, and compensate for the shortcomings of shared development (Ma, 2019), address regional imbalances and inadequacies, and achieve collaborative development across industries. Through cross-regional innovation resource sharing, on the other hand, each innovation resource flows reasonably and orderly and collaborates to support the development of the entire innovation system and promote the effective development of cross-regional resource collaborative innovation activities (Yang et al., 2020). As for the role of synergistic development in influencing shared development, some studies have concluded that synergistic development does not promote shared development, and even sometimes does not contribute to shared development. As a result, this study considers only the role of public goods in influencing synergistic development and proposes the following hypothesis:

*H6*: The relationship between collaborative development and public welfare is influenced positively.

### The impact of high-quality development on the profitability of virtual reality enterprises

Economic development now puts more emphasis on quality, making it obsolete for the previous quantitative evaluation method. According to Ren and Wen (2018), in order to conduct a scientific assessment of quality, one must integrate all indicators and draw conclusions from several viewpoints rather than basing their decision solely on one sign. The quality of economic development has been evaluated by academics from a variety of angles, including the fundamental livelihood aspects of health, fertility, income distribution, political system, religion, and crime. According to Tikhomirov (2019), it is important to determine if economic development is moving from low quality to high quality based on high-tech innovation, when taken as a whole with all economic institutions and social patterns. According to Cao and Feng (2022) financial innovation plays a direct and indirect role in promoting and boosting high-quality regional economic development (Zhang and Zhong, 2021). The five development concepts proposed by China based on high-quality development, namely sharing, coordination, openness, greenness, and innovation, have also been quantified by Yang and Li (2019). Fang and Ma (2019), and others using indicators to assess the level of high-quality development.

At a time when the international economic situation is rapidly changing, the ability of enterprises, as the core component of the national economy, to achieve superior competitiveness in the domestic and even international arenas is dependent on their ability to achieve high-quality development, and high-quality development is a new development paradigm for enterprises to gain competitive advantage (Li, 2019). Environmental regulations are required for businesses to achieve low pollution, low emissions, high efficiency, and long-term quality development. Dasgupta et al. (2001) proposed that environmental regulation is the push-pull effect of government policies. The government promotes economic entities to reduce emissions and consumption by formulating relevant environmental policies and standards. Environmental regulation has been classified into three major categories by scholars: command-and-control, market-based incentives, and public participation (Zhang et al., 2021). Based on environmental governance, the established literature has conducted in-depth studies on the effects of environmental regulation (Guo et al., 2022), economic development (Kiuila and Peszko, 2006; Song and Wang, 2013; Chen and Chen, 2018); technological innovation (Porter and Linde, 1995; Jie et al., 2014); industrial structure optimization (Yuan and Xie, 2014); enterprise-scale (Sun and Wang, 2014); and so on, it has a certain theoretical foundation.

The literature closely related to this study focuses on the impact of environmental regulation on economic development from the standpoint of economic development. On the one hand, some scholars argue that environmental regulations, based on the “neoclassical” viewpoint, can increase firms’ production costs, take up capital, and reduce firms’ competitiveness (Chintrakam, 2008), thereby reducing firms’ performance, which is detrimental to economic development (Barbera and Mcconnell, 1990). Environmental regulation, on the other hand, according to scholars represented by Porter (1991), may increase the cost of emissions and take away Research and Development (Hereinafter referred to as R&D) funds in the short run, but in the long run, environmental regulation will force enterprises to strengthen R&D and innovation in order to save production costs and thus promote economic development. The Porter Hypothesis was proposed and drew the attention and discussion of scholars. Through empirical investigation, scholars such as Chen (2018) proposed that government environmental governance can effectively reduce pollution, which has a catalytic effect on high-quality economic development. He (2018) investigated the impact of environmental regulation on the quality of economic growth from the perspectives of green development and social welfare. The VRI seeks higher social, economic, and environmental benefits through the use of next-generation information technologies such as artificial intelligence, big data, cloud computing, blockchain, and 5G (Chen, 2018). This is an important feature of high-quality development at the current stage, in which enterprises meet the needs of consumers in the new era through high-quality products or services in order to increase profitability and achieve sustainable development. It can be seen that the concept of high-quality development is fully implemented in enterprise production and operation processes, and environmental regulations can be used to compensate for the loss caused by the cost investment in high-quality development. As a result, the following hypothesis is proposed in this study:

*H7-1*: Innovative development has a positive relationship with corporate profitability.

*H7-2*: Green development has a positive relationship with corporate profitability.

*H7-3*: Open development has a positive relationship with corporate profitability.

*H7-4*: There is a positive relationship between public welfare development and corporate profitability.

*H7-5*: Collaborative development has a positive relationship with corporate profitability.

## Research methodology design

This study firstly designs an evaluation system for the high-quality development of VRI based on the perspective of environmental regulation, and uses the entropy-weighted TOPSIS method to measure the collected and collated data to answer the Q1 and Q2 in this paper; secondly, after the initial understanding of the high-quality development of VRI, the profitability of VR enterprises is introduced into the SEM model to explore the Q3 of this paper.

### Virtual reality industry‘s high-quality development indicators measure

To evaluate the relationship between an enterprise’s business situation and high-quality development, a high-quality development evaluation system is required first. Based on the five basic concepts of high-quality development and the characteristics of the VRI (Wei and Li, 2018), this study selects 5 primary indicators and 13 secondary indicators from the China Stock Market and Accounting Research (Hereinafter referred to as CSMAR) Database and Choice financial terminal, as shown in Table 1. The indicator system is primarily based on the logical starting point of the environmental regulation perspective.

**Table 1:** High-quality development evaluation index system.

Tier 1 indicators	Secondary indicators	Indicator description
Innovative development	R&D investment	R&D expenses as a percentage of expenditures on investing activities (+)
	R&D output	Intangible assets/total assets (+)
Green development	Environmental disclosure	Whether to disclose environment and sustainable development (+)
	Energy consumption expenditure	Energy expense overhead as a percentage of revenue (+)
Open development	Degree of separation of powers	Difference between ownership and control (+)
	Supply sources	Percentage of business from top five suppliers (+)
	Sales channels	Business share of top five customers (+)
Public welfare	Donation expenses	External donations as a percentage of revenue (+)
	Public interest disclosure	Whether to disclose public interest information (+)
	Social responsibility Disclosure	Whether to disclose information on social responsibility system construction (+)
Collaborative development	Government relations	Government grants/total revenue (+)
	Corporate relations	The logarithm of the number of related parties (+)
	Cluster relationships	Is in a cluster area (+)

#### Innovative development

The impact of innovation on enterprise development does not happen overnight, and a large amount of capital investment is required in the short term for product and technology development, as well as government regulatory support. According to the perspective of environmental regulation and Porter’s hypothesis, reasonable environmental regulation can encourage enterprises to invest more in innovation and compensate for environmental costs by improving productivity, which is conducive to the rapid transformation of scientific and technological achievements into productivity (Ma et al., 2011). In the long run, technological innovation will transform the entire economic growth mode, as every industrial and technological revolution in history has demonstrated. As a result, both R&D input and R&D output are used as indicators in this study to assess the quality of enterprise innovative development.

#### Green development

In recent years, governments have vigorously advocated green development and actively promoted the conversion of the resource factor-driven economic development model to a low-carbon and sustainable development approach (Li, 2019), and environmental regulation, as governmental regulation, has an innate green development orientation. The discussion of high-quality development at the enterprise level is predicated on the fact that enterprises will prioritize national economic benefits in the production and operation processes. It is frequently stated in project benefit evaluations that national economic benefits should take precedence over financial benefits, so green development of enterprises is based on their ability to reduce environmental costs (Feng et al., 2017). Generally speaking, the VRI harms the environment primarily during the equipment manufacturing process, and the enterprise’s value chain position can be roughly divided by green development as an indicator. Based on environmental regulation and sustainable development theory, each enterprise performs its environmental management duties in accordance with relevant government regulations and standards, and compensates for and reduces environmental costs by optimizing structures and improving innovation, which helps to improve the level of green development and promote long-term enterprise development. As a result, the index system includes the enterprises’ attitude toward green development, and the enterprises’ expenditure on energy consumption is included as one of the evaluation indicators for the disclosure of environmental and sustainable development.

#### Open development

Enterprises must have channels to grow, whether it is through procurement, sales, or investment and financing. Environmental regulation, based on the “pollution sanctuary hypothesis,” attracts capital, industries, and projects that favor ecological and environmental construction by establishing dynamic market access thresholds to increase the level of open development of enterprises (Yuan et al., 2021). Wu et al. (2018) discovered that increasing openness can significantly increase total factor productivity. Because openness involves a variety of economic entities associated with the enterprise, its indicators are based on the evaluation of the enterprise’s stakeholders, such as the enterprise’s equity structure, supplier sources and the number of customer sources, the proportion of foreign investment, and the enterprise’s open development level, which is measured by the width of the enterprise’s channels.

#### Public welfare

This study substitutes public welfare for shared development because shared development includes not only the sharing of economic outcomes but also the sharing of political rights, social security, spiritual culture, and so on. This concept of sharing cannot be used directly as a requirement for enterprises, and even basic sharing of economic outcomes is contrary to the wishes of enterprises. Because public welfare is consistent with the spirit of shared development and can be used as a requirement and evaluation standard for businesses, it is used as a substitute. In addition to the requirements of green development, public welfare reflects enterprise social responsibility. According to environmental regulation and government regulation of public welfare theory, environmental resources as a public resource, through direct intervention of government regulation on economic subjects, can alleviate the unreasonable allocation of resources and the unfairness of distribution, promoting the fulfillment of corporate social responsibility, enhancing social welfare, achieving the goal of protecting environmental resources, and achieving environmental governance (Zhang B, 2020). Enterprise public welfare development can help relieve government pressure and meet people’s needs, which is consistent with the ultimate goal of high-quality development. As a result, the public welfare evaluation system of this study is based on the disclosure of external donations, public welfare information, and information on the construction of the internal social responsibility system of enterprises, which reflect the actual occurrence of public welfare matters and public welfare awareness, as well as whether the internal social responsibility system of enterprises has been established.

#### Collaborative development

It is difficult to achieve development goals on one’s own, and when a company has the same interests as other units, the important way to achieve the goals is to take what they need and develop in a coordinated way. When collaboration becomes the normal development of enterprises, the relationship resources of enterprises in the region become particularly important. Environmental regulation, based on the “collaborative principle” promotes the optimization of industrial structure and industrial transformation through policy pressure, promotes the development of industrial coordination, and sustains enterprise collaborative. Because related parties are the most complex unit of interest for enterprises, they are the primary target of collaborative development between enterprises. Simultaneously, because regional-scale industrial clusters are more conducive to lowering the cost of collaboration, whether an enterprise is located in a cluster region is used as an evaluation index for collaborative development.

### Data processing

First, the CSMAR database was used to collect and organize data from VR concept listed companies over the last 10 years, and then the Choice financial terminal was used to determine the sample of VR concept listed companies involved and whether the companies had a cluster relationship. Second, the sample data were standardized (Wei and Li, 2018), and the processed data were weighted for each index using the entropy weighting method to reduce human interference factors in the assignment process (Yu et al., 2020), and the TOPSIS method was used to measure and rank the high-quality development level of each enterprise. The specific measurement steps are as follows.

Step 1: The data are dimensionlized and the negative indicators are inverted:


(1) $$Y_{ij}=\left\{\begin{array}{c} \frac{x_{ij}-\min\left(x_{ij}\right)}{\max\left(x_{ij}\right)-\min\left(x_{ij}\right)}\\ \frac{\max\left(x_{ij}\right)-x_{ij}}{\max\left(x_{ij}\right)-\min\left(x_{ij}\right)} \end{array}\right.$$

In the equation, *i* denotes an enterprise, *j* denotes a measure, $x_{ij}$ and $Y_{ij}$ denote the original and dimensionless values of the enterprise quality development level measures, $\max\left(x_{ij}\right)$ and $\min\left(x_{ij}\right)$ denote the maximum and minimum values, respectively.

Step 2: It is to calculate the information entropy $E_{j}$ of each indicator $Y_{ij}$:


(2) $$E_{j}=\ln\frac{1}{n}\overset{n}{\underset{i=1}{\sum }}\left[\left(\frac{Y_{ij}}{\sum_{i=1}^{n}Y_{ij}}\right)\ln\frac{Y_{ij}}{\sum_{i=1}^{n}Y_{ij}}\right]$$

Step 3: Weights $W_{j}$ are calculated for each indicator $Y_{ij}$:


(3) $$W_{j}=\left(1-E_{j}\right)/\overset{m}{\underset{j=1}{\sum }}\left(1-E_{j}\right)$$

Step 4: The weighting matrix R of each enterprise’s high-quality development indicators is constructed:


(4) $$R={\left(r_{ij}\right)}_{n\times m}$$

Among them, $r_{ij}=W_{j}\times Y_{ij.}$

Step 5: Determine the optimal solution $Q_{j}^{+}$ and the inferior solution $Q_{j}^{-}$ based on R:


(5) $$\begin{array}{c} Q_{j}^{+}=\left(\max\;r_{i1},\max\;r_{i2},\ldots ,\max\;r_{im}\right)\\ Q_{j}^{-}=\left(\min\;r_{i1},\min\;r_{i2},\ldots ,\min\;r_{im}\right) \end{array}$$

Step 6: Calculate the Euclidean distance $d_{i}^{+}$ and $d_{i}^{-}$ between each measure and the optimal $Q_{j}^{+}$ and inferior solutions $Q_{j}^{-}$:


(6) $$\begin{array}{c} d_{i}^{+}=\sqrt{\sum_{j=1}^{m}{\left(Q_{j}^{+}-r_{ij}\right)}^{2}}\\ d_{i}^{-}=\sqrt{\sum_{j=1}^{m}{\left(Q_{j}^{-}-r_{ij}\right)}^{2}} \end{array}$$

Step 7: The relative proximity $C_{i}$ of each measurement scheme to the ideal scheme is calculated:


(7) $$C_{i}=\frac{d_{i}^{-}}{d_{i}^{+}+d_{i}^{-}}$$

**Figure 1:**
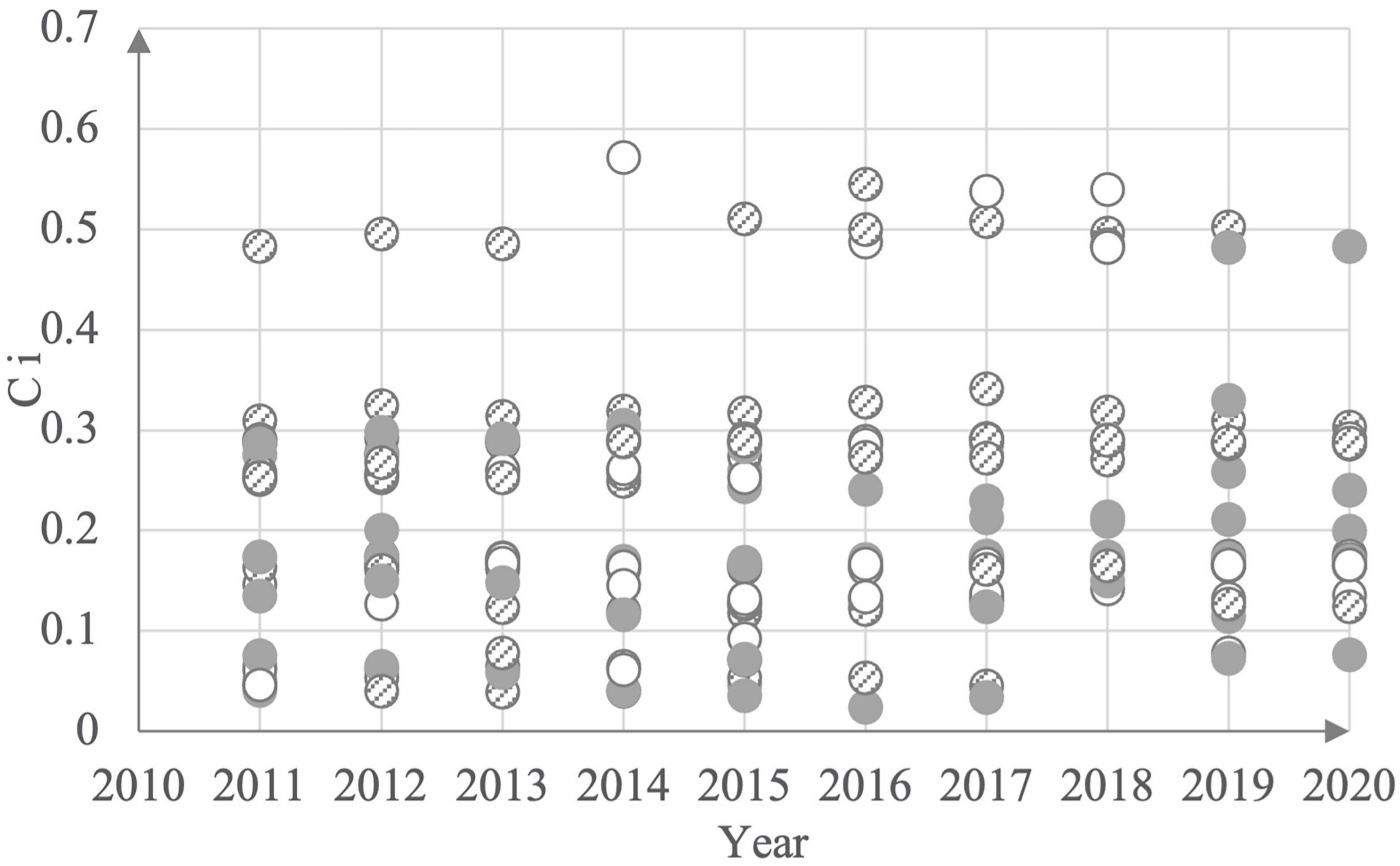
Some VR companies 2011–2020 high quality development level measurement table.

The relative proximity $C_{i}$ indicates the proximity of the evaluation object to the optimal solution and reflects the high-quality development level of the enterprise, and the value is between 0 and 1. The larger the value of C indicates the closer to the optimal solution, i.e., the closer to 1 indicates the higher the high-quality development level of enterprise *i*, and vice versa, the lower the high-quality development level of enterprise *i*. Finally, after removing missing values, the effective sample size is 262, and the measurement results and mean levels are shown in Figure 1 and Table 2.

**Table 2:** Average value of high-quality development in the past 10 years.

Deadline	2011	2012	2013	2014	2015	2016	2017	2018	2019	2020	Total
Average value	0.193	0.207	0.203	0.205	0.190	0.217	0.214	0.258	0.219	0.212	2.118
Number of samples	22	24	24	25	27	28	28	28	28	28	262

From the measurement results, China’s VR listed companies adapt to high-quality development. Although there is still a certain distance from the “high” standard, since 2016, the number of enterprises with a relative proximity Ci of more than 0.4 has increased significantly, and the number of enterprises with a relative proximity Ci of less than 0.2 has decreased significantly. Moreover, the average value of the enterprises has shown an upward trend. Therefore, it can be seen that the degree of high-quality development is constantly rising.

## Empirical analysis

### Analysis of structural equation model construction

Following a preliminary assessment of the VRI’s high-quality development, the relationship between the level of high-quality development and the profitability of the VRI was combined to investigate the path of the role of high-quality development in the VRI. Profitability is defined as an enterprise’s total net asset margin, which is calculated by dividing net profit by the average balance of total assets. Since SEM is suitable for situations with many variables and complex relationships, more abstract and difficult to directly observe attributes can be included in the analysis, and the two steps of principal component regression can be handled at once to reduce the accumulation of statistical errors by reducing the volatility of parameters and predictions.

**Figure 2:**
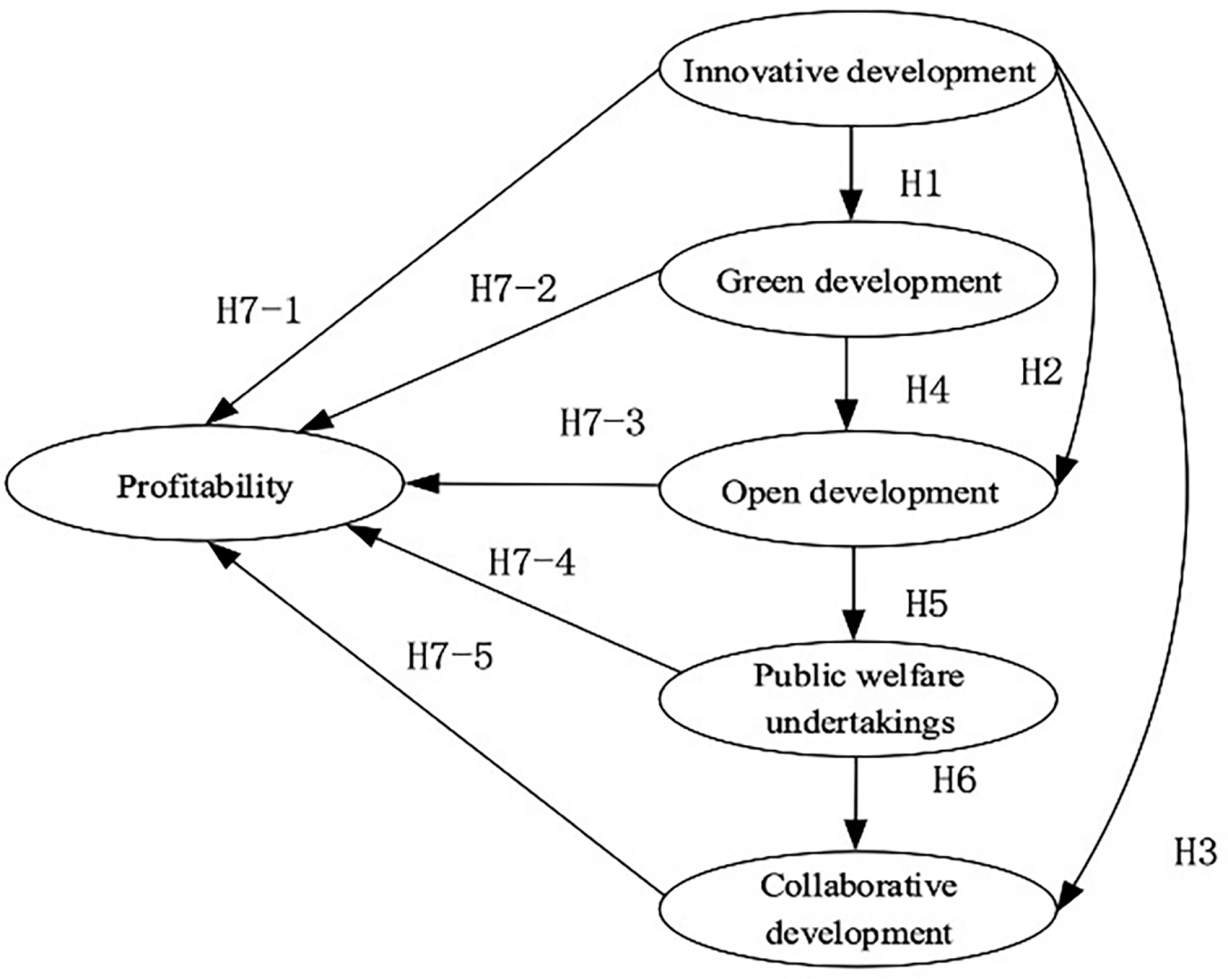
Framework of the relationship between the dimension of high quality development and corporate profitability.

Therefore, SEMs were established to obtain path diagrams to express the relationships among variables, where the variable path relationships were established in one-to-one correspondence with the presupposition of the interaction and influence relationships between latent variables, using innovation development, green development, open development, public welfare, and collaborative development as the five dimensions for evaluating high-quality development. As a result, this study summarizes and compares previous research on high-quality development and conducts an *a priori* analysis based on the following 11 relevant hypotheses, in order to establish the SEM framework of the impact mechanism of high-quality development in the VRI on enterprise profitability. As illustrated in Figure 2.

### Model goodness-of-fit test

Based on the above analysis of the data of listed companies of VR concept from 2011 to 2020, a structural equation model was constructed using AMOS 24.0 software to test the hypotheses presented in the previous section. Table 3 shows the results of the model fit assessment, and six fit indices of the goodness-of-fit test (x^2^/df), goodness-of-fit index (GFI), adjusted goodness-of-fit index (AGFI), normative fit index (NFI), incremental fit index (IFI), and comparative fit index (CFI) were selected to judge the model fit. The results are shown in Table 3.

**Table 3:** Evaluation of model fit.

Fitting index	x^2^/df	GFI	AGFI	NFI	IFI	CFI
Judgment criteria	<5	>0.8	>0.8	>0.9	>0.9	>0.9
Actual value	9.118	0.889	0.767	0.230	0.252	0.215

From the current results, the fitting coefficients of the model are still far from the ideal fit. Therefore, based on the parameter change (PC) and the preliminary estimation of the model’s Modification Indices (M.I.), the observed variables with strong covariance were identified and correlated. After performing the full model path analysis, the possible relevant paths are re-estimated and the revised model is obtained after nested model comparison analysis. The corrected model data achieved a more desirable parameter fit. The correction result is shown in Table 4.

As shown in Table 4, x^2^/df is less than 5; GFI is greater than 0.9; AGFI is greater than 0.8; NFI is 0.974, which is greater than 0.9; IFI is 0.991, which is greater than 0.9; CFI is 0.989, which is greater than 0.9.

**Table 4:** Modified model fit.

Fitting index	x^2^/df	GFI	AGFI	NFI	IF	CFI
Judgment criteria	<5	>0.8	>0.8	>0.9	>0.9	>0.9
Actual value	1.543	0.996	0.959	0.974	0.991	0.989

The model fit test indexes all reach the ideal value, indicating that the model fit is satisfactory. This indicates that the latent variables in the constructed model provide good explanations for the relationship between high-quality VRI development and enterprise profitability, and thus the model fit is judged to meet the test requirements.

**Figure 3:**
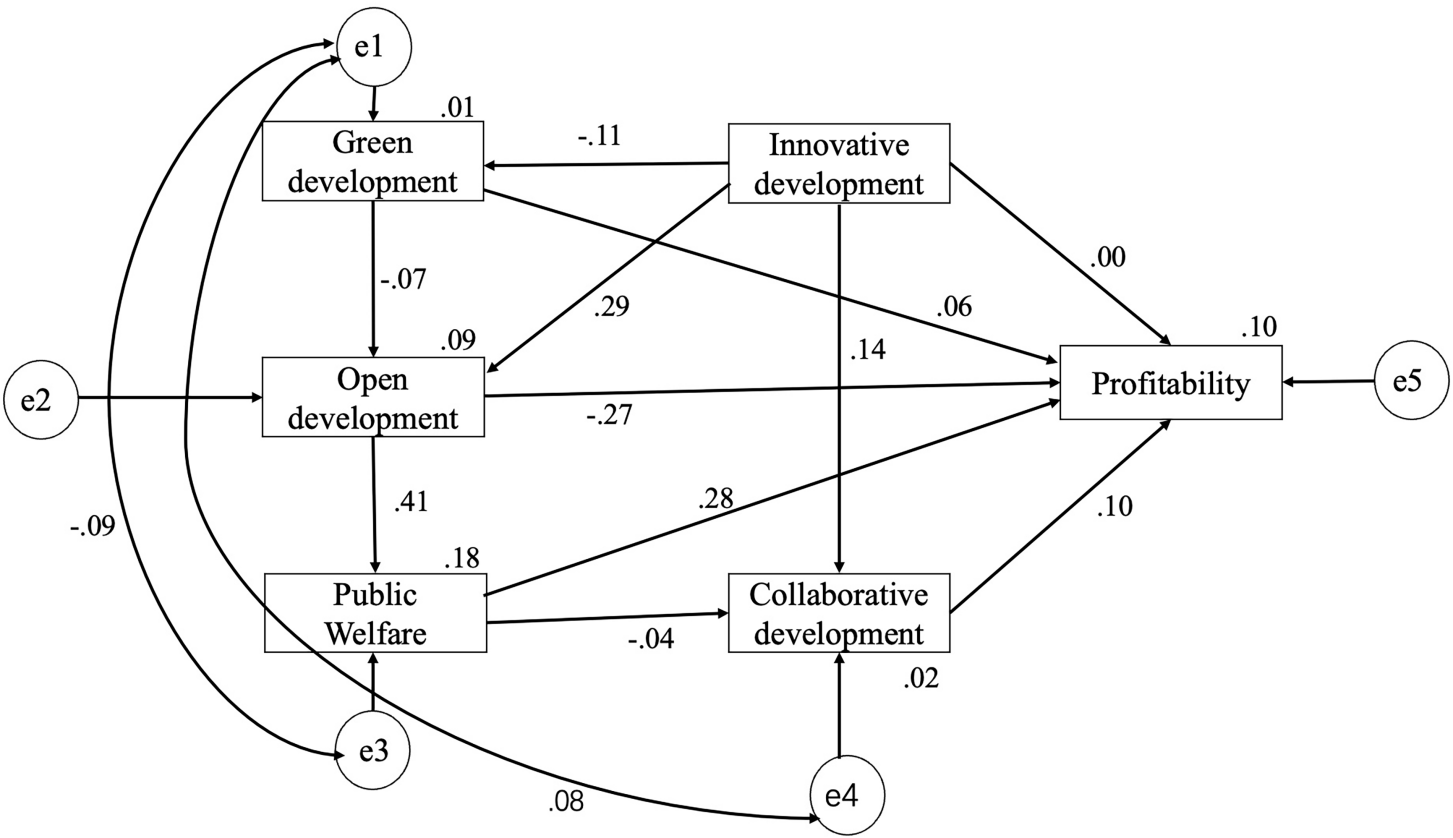
Calculation results of modified structural equation model.

The final modified model output is shown in Figure 3.

### Hypothesis testing and results analysis

The previous section’s hypotheses were tested further, and the results are shown in Table 5: innovation development has a negative impact on green development, and hypothesis 1 was not verified. Innovation development has a positive impact on both open and coordinated development, with open development having a larger impact (=0.289, *p* < 0.001), and hypotheses 2 and 3 are proved. In China, the VRI is still in its early stages; there are opportunities and challenges; enterprises’ technological innovation and competitiveness have increased; and there is a “closed door development” phenomenon. The VRI is not yet a mature industrial cluster. On the one hand, it lacks industry norms, and on the other hand, it neglects its responsibilities to the environment and society.

**Table 5:** Hypothesis testing results.

Assumptions	Structural model	Path factor	*p*-value	Hypothesis testing
H1	Innovative development → Green development	−0.108	0.079	Not supported
H2	Innovative development → Open development	0.289	***	Support
H3	Innovative development → Collaborative development	0.136	0.028	Support
H4	Green development → Open development	−0.068	0.252	Not supported
H5	Open development → Public welfare	0.414	***	Support
H6	Public welfare → Collaborative development	−0.045	0.470	Not supported
H7-1	Innovation and development → Profitability	−0.003	0.966	Not supported
H7-2	Green Development → Profitability	0.055	0.353	Not supported
H7-3	Open development → Profitability	−0.274	***	Not supported
H7-4	Public welfare → Profitability	0.278	***	Support
H7-5	Collaborative development → Profitability	0.102	0.086	Support

*** indicates *p* < 0.001.

Green development has a negative impact on open development, and Hypothesis 4 has not been verified. The impact of open development on public welfare is positive and significant (=0.414, *p* < 0.001), and hypothesis 5 is proved. The VRI’s open development means more channels for customer-supplier relationships in social networks, as well as a greater need to form social norms to achieve shared development. Hypothesis 6 was not proven because public welfare has a negative impact on collaborative development.

Public welfare development and coordinated development in high-quality development have a positive impact on the profitability of enterprises, with public welfare development having the greatest impact, and hypotheses H7-4 and H7-5 are proved. Innovative development has a negative impact on profitability, while green development has a positive impact on profitability, but the impact is not significant. Hypotheses H7-1 and H7-2 are not proved. Open development has a significant negative impact on profitability (= −0.274, *p* < 0.001), and hypothesis 7-3 is not supported. When the VRI expands channels and scales, it consumes more energy in the short term, loses core competitiveness, and makes it difficult for companies to be profitable. The findings indicate that there is a significant interaction between green development and public welfare and collaborative development, with the main mechanism of action being positive feedback between public welfare and enterprise profitability. A multi-level collaborative of factors is required to achieve high-quality development of the VRI. The calculation results of the modified SEM and the influence effects among the variables are shown in Table 6.

**Table 6:** Effects among the variables.

Impact effect	Profitability		Innovative development	Green development	Open development	Public welfare
Innovative development	Path factor	−0.003	-	-	-	-
	Indirect effects	−0.054	-	-	-	-
	Total effect	−0.057	-	-	-	-
Green development	Path factor	0.055	−0.108	-	-	-
	Indirect effects	0.011	-	-	-	-
	Total effect	0.066	−0.108	-	-	-
Open development	Path factor	−0.274	0.289	−0.068	-	-
	Indirect effects	0.113	0.007	-	-	-
	Total effect	−0.161	0.296	−0.068	-	-
Public welfare	Path factor	0.278	-	-	0.414	-
	Indirect effects	−0.005	-	-	-	-
	Total effect	0.273	-	-	0.414	-
Collaborative development	Path factor	0.102	0.136	-	-	−0.045
	Indirect effects	-	−0.006	-	-	-
	Total effect	0.102	0.130	-	-	−0.045

## Conclusion and discussion

### Discussion

Studies have shown that economic development (Kiuila and Peszko, 2006; Song and Wang, 2013; Chen and Chen, 2018); technological innovation (Porter and Linde, 1995; Jie et al., 2014); industrial structure optimization (Yuan and Xie, 2014); enterprise-scale (Sun and Wang, 2014) have a positive impact on the development of industry, for example, environmental regulation through the development of environmental regulatory tools such as green entry barriers that will stimulate enterprises to improve their innovation capabilities, improve the efficiency and quality of green product production, and promote the transformation and upgrading of industrial structure (Li and Nie, 2009; Yu and Wu, 2022).

However, the path of environmental regulation on high-tech industries is under-researched. From the perspective of high-quality development goals, the widespread use of VR technology can meet the spiritual civilization needs of users, which is consistent with the goal of improving people’s quality of life. According to some scholars, the VRI is distinguished by a broad range of industry chain coverage and strong policy support, as well as a high level of industry clustering and low barriers to industry entry, heralding abundant opportunities while confronting enormous challenges. The combination of these characteristics demonstrates that VR enterprises have a large-scale social relationship network, rich potential resources, a high industry reputation, and close cooperation among all links of the industry chain, and can steadily improve development quality with environmental regulation as a foundation.

### Conclusion

This study uses entropy method and SEM model to explore the role path and evolutionary mechanism of high-quality development of VRI and reveals the impact of high-quality development on the profitability of VRI. In particular, following the measurement of the level of high-quality development in the VRI, the hypothesis of the main influence of high-quality development on the profitability of the enterprise itself is presented, and the influence mechanism and path of action of high-quality development in the VRI on the profitability of the enterprise are empirically tested.

According to the model’s calculation results, we know that: first, the five evaluation dimensions mostly have a direct effect on enterprise profitability, but also indirectly through the mutual influence of the dimensions. Public welfare development has the greatest impact on corporate profitability, and it also has the greatest direct impact on corporate profitability. Second, open development influences enterprise profitability through public welfare development. With the implementation of a national policy of opening up to the outside world, “bringing in” and “going out” can result in long-term profitability for businesses. Third, the degree of green development and coordinated development has a minor impact on enterprise profitability. In general, as the basic concept of high-quality development, low-carbon and sustainable development can help enterprises achieve profitability; collaborative development is the development goal of the VRI chain, which ultimately helps enterprises achieve high-quality development. Fourth, there is a negative relationship between the innovation dimension and enterprise profitability, indicating that in recent years, innovation development has caused enterprises to increase a large amount of R&D investment in innovation, which has weakened their profitability level; however, the degree of this negative impact is small, and in the long term, innovation development can reduce enterprise resource consumption through technological progress. Overall, high-quality development does not benefit all VR enterprises, so there is still room to improve high-quality development.

In summary, we find that all five evaluation dimensions directly or indirectly affect the profitability of VR enterprises, filling the gap in the study of the development level of high-tech industries from the perspective of environmental regulation. However, considering the availability of data and the fact that the development of China’s VRI is in its infancy and most VR enterprises have injected state capital at different times, this study does not do heterogeneity analysis and focuses only on the financial performance of the VRI. Future research work can combine more perspectives for analysis or collect information about the VRI in multiple dimensions through other methods to expand the research.

## Related recommendations

This research provides some targeted suggestions for the China’s VRI and related industries in foreign countries:

Business operations are primarily concerned with realizing economic interests, and high environmental costs are one of the major barriers to high-quality development for VR companies. VR enterprises can consider the environmental regulation cost to be attributed to the supply source and included in the homemade cost before deciding on outsourced or homemade raw materials. Including supplier selection, such as considering environmental benefits of suppliers, on the one hand to avoid the transfer of environmental taxes, and on the other hand to benefit enterprises’ social reputation. Maintaining good relationships with customers and suppliers facilitates the flow of information and lowers transaction costs. The most direct way for VR enterprises to protect the environment is not to increase investment in environmental management, but to control and reduce the consumption of environmental resources in order to achieve sustainable development, both to control costs and to maintain the stock of social capital.

Recruiting the upstream enterprises of the supply chain to settle in the cluster. The cluster organization and management construction will promote the development of SMEs through the demonstration of leading enterprises, provide supporting facilities, and carry out the backward integration strategy for the downstream industries, forming a mutually complementary ecosystem of large and medium-sized enterprises, which is conducive to accelerating the upgrading of the VRI chain.

The overall green development level of the VRI in general, innovation capacity is also relatively weak; as a high-tech industry, there is still a gap from high-quality development; if we want to achieve high-quality development, we must improve our resource utilization and innovation capacity. Enterprises can fully utilize social capital’s resourcefulness to integrate resources and realize an effective combination of internal and external resources. On the one hand, businesses should appropriately reduce work pressure on employees, strengthen corporate culture construction so that employees have a sense of belonging and happiness, and encourage technological innovation; on the other hand, enterprises can strengthen collaboration with universities or other correspondent units, deepen collaboration between government, industry, and academia, fully utilize external resources within the geo-environment, improve their innovation capacity, and lay a solid technical foundation for moving up the global value chain.

The majority of VR companies are committed to content production, and their products are in the low-end market. The VRI is still in a bubble period, and the number of manufacturers flooding the content market is massive, becoming the main concentration of the bubble. To make supply products and improve core competitiveness, each enterprise should clarify its own industry needs, fully understand the various levels of industry needs, and improve its core competitiveness. While realizing their interests and ensuring product quality, enterprises should focus on social value and actively assume social responsibility, as guided by the requirements of high-quality development and the concept of development in the new era, which will help increase the stock of social capital and the formation of a good corporate image, and lay the global value chain status.

VR is a new generation of information technology and is recognized as the next windfall of the information industry. The global VRI is moving from the initial cultivation period to the rapid growth period. In the face of development opportunities, various forms of international exchange and cooperation, we can create a good development environment and promote the interaction of technology, talents, capital, and other resources to achieve open sharing and mutual benefit.

## Theoretical and practical implications

The main theoretical and practical implications of this study are as follow.

First, this study is based on an environmental regulation perspective, combining the entropy method and the SEM to broaden the research breadth of enterprise high-quality development. At the moment, most research on the level of high-quality development of industries focuses on traditional industries, with little research on emerging industries, which are mostly analyzed from the standpoint of innovation development, ignoring the importance of green development level. This study incorporates environmental regulation theory into the variable model, analyzes the high-quality development level from the standpoint of environmental regulation, and uses the high-quality development level measure to investigate the VRI’s applicability to various industries.

Second, it provides a model for the development of policies to promote the high-quality development of the VRI, which, as a key development type of the digital economy, is critical to economic development. This study assists the government in formulating improved policies that are in line with the development of the industry and the improvement of the enterprises’ development strategies by analyzing the development status of the VRI and exploring the impact mechanisms between the high-quality development of the VRI and the profitability of each enterprise.

In addition, it provides a reference for the high-quality development of foreign VRI. The global VRI is moving from the initial cultivation period to the rapid growth period. China is one of the regions with the most active innovation and entrepreneurship, the highest market acceptance, and the greatest development potential in the global VRI. Facing the development opportunities, China will further increase its efforts to promote the high-quality development of the VRI, and the experience of China’s VRI development can provide new ideas for the industrial development of other countries. In addition, 71% of China’s VR market share belongs to the enterprise market and 29% to consumers. In other countries, the opposite is true: 70% of the market share is for consumers and 30% for enterprises. The early development of technology will go through the process of enterprise to the consumer level, and the VRI is relatively niched at the beginning of development, but the future can enter thousands of households. Therefore, foreign countries can learn from China’s VRI development path to adjust its development direction or broaden the VRI in the enterprise market share.

## Data availability statement

The original contributions presented in the study are included in the article/Supplementary Material, further inquiries can be directed to the corresponding author.

## Author contributions

LZ proposed a research topic, obtained research funds, and reviewed and revised the thesis. YY was responsible for researching and organizing literature, wrote the first draft of the thesis, and validated and verified the experimental design. KX collected and organized data and visualized the experimental results. All authors contributed to the article and approved the submitted version.

## Funding

This work was supported by National Natural Science Foundation of China (72262014 and 62261023), Jiangxi Provincial Social Science Planning Project (21GL15), and Jiangxi Provincial Humanities and Social Science Project for Universities (GL21114).

## Conflict of interest

The authors declare that the research was conducted in the absence of any commercial or financial relationships that could be construed as a potential conflict of interest.

## Publisher’s note

All claims expressed in this article are solely those of the authors and do not necessarily represent those of their affiliated organizations, or those of the publisher, the editors and the reviewers. Any product that may be evaluated in this article, or claim that may be made by its manufacturer, is not guaranteed or endorsed by the publisher.
